# Assessing the Prognostic Accuracy of Red Cell Distribution Width for Predicting Mortality in Sepsis Patients: A Comparative Study With Serum Lactate and the Quick Sequential Organ Failure Assessment (qSOFA) Score

**DOI:** 10.7759/cureus.80056

**Published:** 2025-03-04

**Authors:** Sweety Singh, Niraj Diwakar, Rishabh Jha, Prachi Vatsa, Arshad Ahmad

**Affiliations:** 1 Internal Medicine, Lady Hardinge Medical College, New Delhi, IND; 2 Internal Medicine, Indira Gandhi Institute of Medical Sciences, Patna, IND; 3 General Medicine, Indira Gandhi Institute of Medical Sciences, Patna, IND

**Keywords:** lactate, mortality, qsofa, red cell distribution width, sepsis

## Abstract

Background: Sepsis is a clinical condition characterized by high morbidity and mortality. Several prognostic markers have been evaluated to identify patients with severe sepsis. The red cell distribution width (RDW) is a cost-effective and widely accessible laboratory test to predict mortality across various medical conditions. This research aimed to evaluate the role of RDW as a predictor of mortality in sepsis patients and compare its predictive value with that of serum lactate levels and the qSOFA (quick Sequential Organ Failure Assessment) score.

Methods: This prospective observational study enrolled sepsis patients admitted to the Medicine Department at the Indira Gandhi Institute of Medical Sciences between March and August 2024. The study evaluated the discriminatory ability of RDW, serum lactate levels, and qSOFA score for predicting mortality by calculating the ROC-AUC (receiver operating characteristic area under the curve).

Results: Out of 100 eligible patients, 97 had been incorporated into the final analysis. The mean RDW was significantly elevated among the non-survivors, and it had been recognized as an independent predictor of 30-day mortality (OR=2.91, (1.56; 5.42), p < 0.001). For predicting 30-day mortality, the AUC values were as follows: RDW at 0.878 (p < 0.001), lactate at 0.719 (p < 0.001), and qSOFA score at 0.837 (p < 0.001). The cutoff value for RDW was established at 14.15%.

Conclusion: RDW was significantly associated with 30-day mortality in sepsis patients and was found to be an independent prognostic marker for predicting mortality. Its mortality discriminative ability was comparable to that of the qSOFA score but superior to that of lactate.

## Introduction

Sepsis is a critical medical illness that results in significant morbidity as well as mortality. It results from an exaggerated immune response to infection [1] and is one of the primary causes of hospital fatalities, with mortality rates of 18.7% among inpatients and 55.7% in ICU (Intensive Care Unit) patients [2]. Sepsis has a high incidence globally, with 31 million sepsis and 19 million severe sepsis cases, leading to five million deaths every year [3]. The fatality rate in India is found to be 213 per 100,000 patients with sepsis [4].

Several biomarkers have been evaluated to predict and diagnose sepsis and guide its management in patients; however, no definitive parameters have been established to date [5]. Commonly used biomarkers for sepsis include procalcitonin levels, lactate, and C-reactive protein (CRP). Commonly employed clinical scoring systems are the qSOFA (quick SOFA) score, SOFA (Sequential Organ Failure Assessment) score, and the APACHE II (Acute Physiology and Chronic Health Evaluation) score.

The red cell distribution width (RDW) quantifies the variability in dimensions of circulating erythrocytes, ascertained through a complete blood count (CBC). It exhibits the dysregulation of iron metabolism and inhibition of erythropoiesis, resulting in anemia of chronic disease, mediated by diverse cytokines, primarily IL-6 [6]. In sepsis, oxidative stress and inflammation are critical factors that can reduce red blood cell survival and impair their maturation [7]. This situation results in the release of immature erythrocytes into circulation, leading to an increase in RDW values. Multiple studies have contemplated RDW as an inflammatory marker or a predictor of mortality in various clinical settings, including infections, chronic inflammatory diseases, cardiovascular disease, and acute respiratory distress syndrome (ARDS). Consequently, RDW might be clinically helpful in identifying patients with sepsis as it is readily available and inexpensive [8]. Therefore, this study is important for evaluating the effectiveness of RDW in predicting mortality in sepsis patients and comparing it with serum lactate levels and qSOFA score.

## Materials and methods

**Figure 1:**
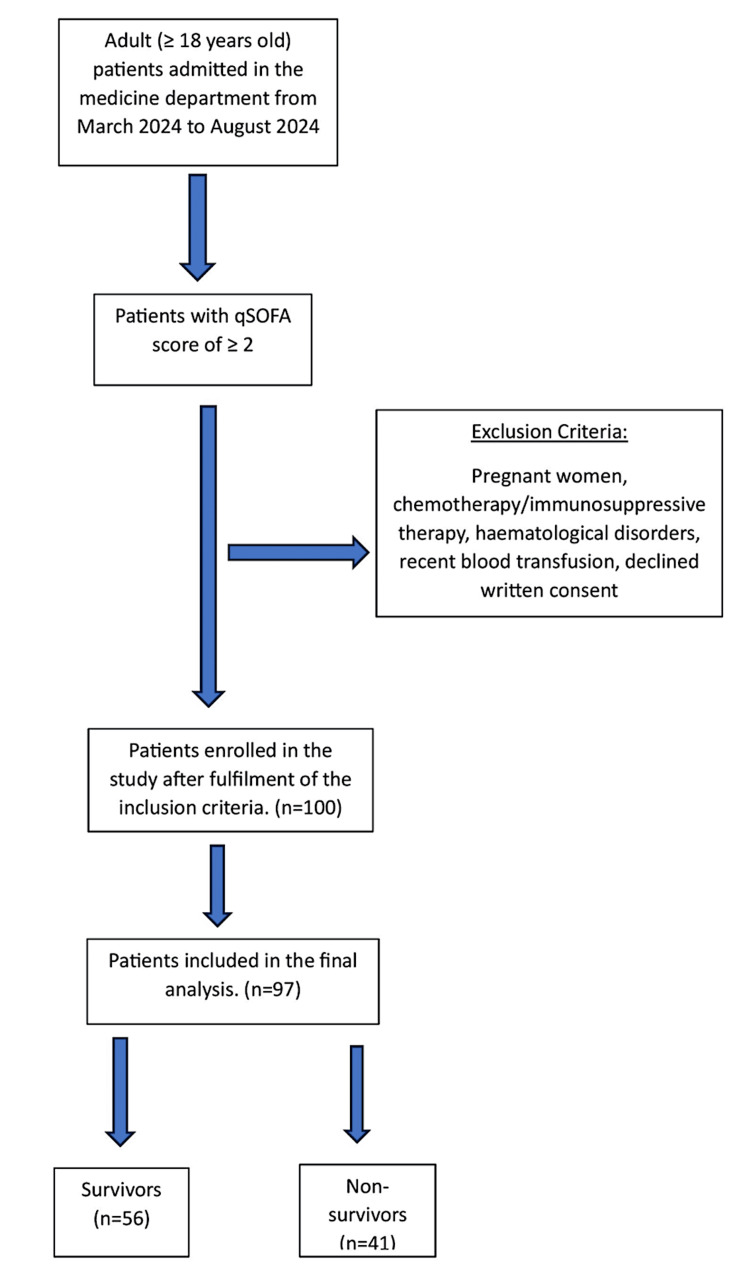
Flowchart of the study qSOFA: quick Sequential Organ Failure Assessment

This prospective observational study was conducted by the Medicine Department at the Indira Gandhi Institute of Medical Sciences from March to August of 2024. It included 100 patients aged over 18 years with a qSOFA score of 2 or more. The qSOFA score varies from 0 to 3, with 1 point allotted for each of the following clinical signs: (1) Glasgow Coma Score of 13 or less, (2) systolic blood pressure of 100 mmHg or lower, and (3) respiratory rate of 22 per minute or more. A score equal to two or more indicated severe sepsis. The exclusion criteria comprised patients who declined written consent, pregnant women, those undergoing chemotherapy or on immunosuppressive drugs or erythropoietin, individuals with known hematological disorders such as leukemia or myelodysplastic syndrome, and patients who had received a blood transfusion within the past week or had experienced recent bleeding (Figure 1).

Data were collected using standardized forms. The primary outcome was defined as all-cause mortality occurring within 30 days of hospital admission. In this study, serum lactate levels were systematically measured within the first 24 hours of patient admission. RDW was recorded during the CBC analysis at admission using a Beckman Coulter automated analyzer (Brea, CA, USA), with a reference value for RDW-coefficient of variation set at 11.6%-14% for the institution. The Institutional Ethics Committee at Indira Gandhi Institute of Medical Sciences (IGIMS), Patna, granted approval for the study.

Continuous data were documented as means with standard deviations, whereas categorical data were expressed as percentages. The study population was categorized into survivors and non-survivors, and comparisons were made between these groups. Numerical variables were assessed using the Student's t-test or Mann-Whitney U test, whereas categorical variables were evaluated with the chi-square test. Key variables were analyzed using multivariable logistic regression to determine the factors linked to hospital mortality. The predictive ability of RDW, lactate, and qSOFA score for mortality was evaluated utilizing ROC (receiver operating characteristic) curves, and a comparison was done by calculating the AUC (area under the curve). Employing Youden's index, the cutoff value for RDW was resolved. A p-value of less than 0.05 was considered statistically significant. Comprehensive statistical analyses were conducted using IBM SPSS Statistics for Windows, Version 25 (Released 2017; IBM Corp., Armonk, New York).

## Results

The study included a total of 100 patients who met the inclusion criteria. Among these, three individuals had been lost to follow-up, leaving 97 for final analysis. The average age of participants was 52.04 ± 17.85 years (range 18-93 years). The total number of male patients was 64.95%, and female patients were 35.05%. The most prevalent site of sepsis at admission was the abdomen (31.95%), followed by the respiratory tract (30.92%), urinary tract (18.55%), central nervous system (7.21%), and skin and soft tissue (6.18%). The most common comorbidity found was diabetes mellitus (28.86%), followed by hypertension (10.30%), stroke (9.27%), cardiovascular disease (7.20%), and pulmonary disease (6.18%). The mean hemoglobin level among the study population was 10.67 ± 2.54 g/dL, and the total leukocyte count was 21.27 ± 8.33×10^3^/cmm. RDW and serum lactate mean values were 16.59 + 2.17 and 2.12 + 1.69, respectively. The mean duration of hospital stay observed was 10.74 ± 5.13 days. The research population's overall mortality rate was 42.2%.

The study population was divided into non-survivors and survivors based on their outcomes. Various parameters, including demographic, clinical, and laboratory data, were analyzed among the two groups (Tables 1, 2).

**Table 1:** Demographic parameters of the study population Test statistics: chi-square (χ²) and t-test values, depending on the variable type. p-value: measures statistical significance, with values <0.05 indicating significant differences between the survivors and non-survivors.

Characteristics	Survivors (n=56)	Non-survivors (n=41)	p-value	Test Statistics
Age, years (mean±SD)	49.75±17.61	55.17±17.91	0.140	1.487
Male gender (%)	38 (67.8%)	25 (60.9%)	0.482	0.492
Comorbidities				
Diabetes mellitus (%)	16 (28.5%)	12 (29.3%)	0.940	0.006
Hypertension (%)	3 (5.3%)	7 (17.1%)	0.061	3.514
Stroke (%)	6 (10.7%)	3 (7.3%)	0.120	2.420
Cardiovascular disease (%)	2 (3.6%)	5 (12.2%)	0.104	2.629
Pulmonary disease (%)	4 (7.1%)	2 (4.9%)	0.647	0.209
Chronic liver disease (%)	3 (5.3%)	2 (4.8%)	0.916	0.011
Others (%)	5 (8.9%)	4 (9.7%)	0.889	0.019
Site of infection				
Abdomen (%)	20 (35.7%)	11 (26.8%)	0.353	0.859
Respiratory tract (%)	17 (30.3%)	13 (31.7%)	0.887	0.020
Urinary tract (%)	12 (21.4%)	6 (14.6%)	0.395	0.723
Central nervous system (%)	3 (5.3%)	4 (9.7%)	0.408	0.684
Endocarditis (%)	1 (1.8%)	2 (4.9%)	0.385	0.755
Skin and soft tissue (%)	4 (7.1%)	2 (4.9%)	0.647	0.209
Bloodstream infection (%)	0	2 (4.9%)	0.095	2.789
Unknown (%)	0	3 (7.3%)	0.040	4.228

**Table 2:** Clinical and laboratory parameters of the study population Test statistics: chi-square (χ²) and t-test values, depending on the variable type. p-value: measures statistical significance, with values <0.05 indicating significant differences between the survivors and non-survivors.

Characteristics		Survivors (n=56)	Non-survivors (n=41)	p-value	Test Statistics
Hemoglobin (g/dL) (mean±SD)		11.28±2.21	9.83±2.72	0.005	-2.873
Total leucocyte count (×10^3^/µL) (mean±SD)		20.84±8.87	21.84±7.60	0.565	0.578
qSOFA (%)	Score 2	50 (89.3%)	9 (22.0%)	-	-
	Score 3	6 (10.7%)	32 (78.0%)	<0.001	45.038
RDW (%) (mean±SD)		15.41±1.39	18.18±2.03	<0.001	7.965
Lactate (mmol/L) (mean±SD)		1.54±0.76	2.90±2.22	<0.001	4.241
Ionotropic support (%)		45 (80.3%)	36 (87.8%%)	0.329	0.953
Ventilatory support (%)		16 (28.6%)	35 (85.4%)	<0.001	30.622
ICU admission (%)		19 (33.9%)	36 (87.8%)	<0.001	27.985
Total stay (days) (mean±SD)		13.41±4.54	7.10±3.33	<0.001	-7.533

The survivor patients had a slightly younger mean age (49.75 years) than the non-survivor group (55.17 years). The most prevalent comorbidity observed in both groups was diabetes mellitus. The most common site of sepsis among survivors was the abdomen, while the respiratory tract was most common among non-survivors. Additionally, three patients (3.09%) had more than one site of sepsis.

Among the non-survivors, the mean hemoglobin level was lower than that of the survivors (p < 0.005). Additionally, the non-survivors had notably greater qSOFA scores compared to the survivors (p < 0.001). The mean RDW was found to be higher in non-survivors (18.18 versus 15.41) with a significant p-value of <0.001. Additionally, the mean serum lactate level was slightly elevated in non-survivors (p < 0.001). ICU admission and the requirement for mechanical ventilation were significantly linked to higher mortality rates (p < 0.001). Furthermore, the duration of hospital stay was longer for survivors compared to non-survivors (p < 0.001).

**Figure 2:**
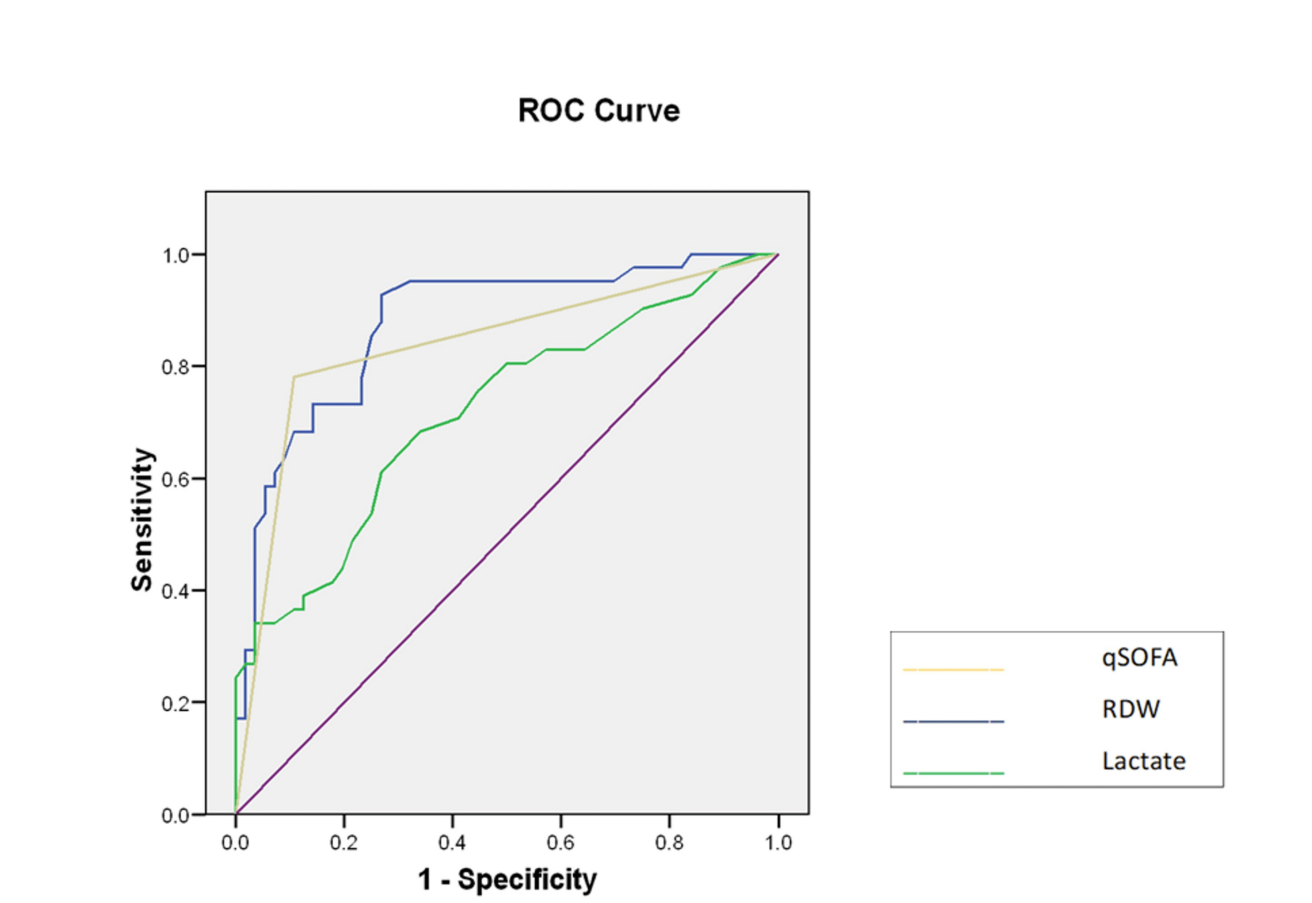
Receiver operator characteristic (ROC) curve for RDW, serum lactate, and qSOFA for 30-day mortality RDW: red cell distribution width; qSOFA: quick Sequential Organ Failure Assessment

A multivariate logistic regression analysis evaluated the relationship between significant factors and 30-day mortality. RDW emerged as the sole independent predictor of 30-day mortality (OR=2.91 (1.56; 5.42), p <0.001). An ROC curve analysis was conducted to assess the discriminatory ability to predict mortality (Figure 2), and the AUC was determined. The AUC for serum lactate was 0.719 (95% CI, 0.615-0.823; p < 0.001), whereas the AUC for the qSOFA score was 0.837 (95% CI, 0.748-0.925; p < 0.001). Table 3 presents the AUC of 0.878 (95% CI, 0.807-0.948; p < 0.001) for RDW, with a cutoff value established at 14.15%.

**Table 3:** AUCs of RDW, serum lactate, and qSOFA AUC: area under the curve; qSOFA: quick Sequential Organ Failure Assessment; RDW: red cell distribution width

Characteristics	AUC	95% Confidence Interval	p-value
RDW	0.878	0.807-0.948	<0.001
Lactate	0.719	0.615-0.823	<0.001
qSOFA	0.837	0.748-0.925	<0.001

## Discussion

This research was designed to examine the role of RDW in predicting mortality among sepsis patients enrolled in the Department of Medicine at the Indira Gandhi Institute of Medical Sciences. RDW is an inexpensive and common laboratory test that measures the degree of variability of RBC size. RDW values are dependent on various factors. Elevated RDW is seen in conditions that lead to either increased destruction or decreased production of RBC. Sepsis is a pro-inflammatory state that often leads to decreased erythropoietin production and reduced iron bioavailability, causing suppressed erythroid precursor activity in the bone marrow. This condition results in the release of premature erythrocytes into circulation, which contributes to elevated RDW levels.

In our study, mortality among the study population was 42.2%. The mean RDW was considerably larger in the non-survivors than that in the survivors (p<0.001). The non-survivors exhibited a higher qSOFA score than the survivors (p < 0.001). The mean serum lactate level was slightly elevated among the non-survivors (p < 0.001). The AUC for the qSOFA score was 0.837 (p < 0.001), 0.719 (p < 0.001) for serum lactate, and 0.878 (p < 0.001) for RDW. RDW was significantly linked to 30-day mortality in patients with sepsis as well as recognized as an independent predictor of 30-day mortality (OR=2.91, (1.56; 5.42), p < 0.001). Its discriminatory ability was comparable to that of the qSOFA score but outperformed serum lactate levels in predicting mortality.

A study by Uffen et al. involving 1,046 patients found that RDW serves as an independent predictor of early clinical deterioration and 30-day death in sepsis patients, achieving an AUROC of 0.66 for forecasting 30-day mortality [9]. In a comparable investigation by Wang and Tsu, patients with high RDW demonstrated a statistically higher rate of ICU admission (p = 0.03), septic shock (p < 0.01), as well as 30-day mortality (p < 0.01). Additionally, RDW (AUC = 0.71) showed superior discriminative ability for predicting mortality compared to lactate levels (AUC = 0.63), which is consistent with our findings [10]. In a study by Jain et al., the RDW value was significantly elevated in patients with severe sepsis and non-survivors compared to survivors (p < 0.0001). A strong correlation was observed between the SOFA score and RDW in predicting disease outcomes, with a Pearson correlation coefficient of r = 0.46. The AUC for RDW was 0.852 at a 95% CI (0.796-0.909), with a cutoff of 17.15 [11]. Conversely, in another study by Jandial et al., RDW was correlated with 30-day mortality but was not established as an independent prognostic indicator for its prediction [7].

This study has several strengths. It was a prospective study; therefore, conditions that could have influenced RDW values were reviewed, such as transfusion history or use of erythropoietin before admission. Patients with such a history were excluded from the study. Blood transfusion is a significant factor contributing to elevated RD, and earlier studies often did not account for this due to their retrospective design. The primary outcome, defined as 30-day mortality, is a widely accepted measure in critical care research. RDW was assessed at admission, ensuring it was not influenced by medical management during the hospital stay.

However, this research has certain limitations. Apart from the small sample size, the study was executed at a single tertiary care center and included only patients from the Medicine Department. Thus, the results may not be generalizable to other healthcare institutions or surgical patients with sepsis.

## Conclusions

RDW demonstrated a significant association with 30-day mortality in sepsis patients and was found to be an independent prognostic marker for its prediction. The mortality discriminative ability of RDW was comparable to that of the qSOFA score but was superior to that of serum lactate. Therefore, RDW can be used as a reliable marker for predicting mortality in patients with sepsis.
